# Corrections in an electronic environment

**DOI:** 10.1186/1741-7015-5-4

**Published:** 2007-03-23

**Authors:** Melissa L Norton, Deborah C Saltman

**Affiliations:** 1 Medical Editor, BioMed Central, 34-42 Cleveland Street, London, W1T 4LB, UK; 2 Editorial Director (Medicine), BioMed Central, 34-42 Cleveland Street, London, W1T 4LB, UK; 3 Discipline of General Practice, University of Sydney, 37A Booth Street Balmain, NSW, 2041 Australia

## Abstract

The need to publish corrections to scientific articles, and occasionally to retract them, has been recognized for decades. However very little emphasis has been attached to how this is done, provided that the retraction or correction is accessible. We are considering a policy to directly correct our online publications.

## 

The need to publish corrections to scientific articles, and occasionally to retract them, has been recognized for decades. However very little emphasis has been attached to how this is done, provided that the retraction or correction is accessible [1]. In a paper-based environment, the location of these corrections has been determined by the ease of finding the correction and its prominence in relation to the published article.

Archivists and librarians are on record in their support of the historical importance of maintaining the publication trail and therefore maintaining the original. As T. Scott Plutchak, Editor of the *Journal of the Medical Library Association*, said, "We must never forget that the preservation of the historical record, with all of its faults, mistakes, and corrections, is an essential part of the service that librarianship performs for society. As the medium of information becomes more elusive, we must become more vigilant" [2].

The underlying implication is that the historical work maintains some intrinsic merit, whether right or wrong. In the rapidly changing environment of electronic publishing, however, one of the key benefits for authors is the capacity to document their work fully and accurately in a timely fashion [3]. When this does not happen, misinterpretation is bound to occur. In one study, 235 retracted articles were cited 2034 times after the retraction notice was posted. Examination of 299 of those citations reveals that in only 19 instances was the retraction noted; the remaining 280 citations treated the retracted article either explicitly (n = 17) or implicitly (n = 263) as though it were valid research [4].

Studies of the biomedical literature have shown that retractions are more than twice as likely to result from unintentional mistakes as from scientific misconduct. [5] Nath *et al*. also suggest that the different characteristics of articles retracted for misconduct and for unintentional mistakes reflect distinct causes and, potentially, point to distinct solutions.

We want to provide our readers with the most accurate presentation of our authors' work. "Dynamic documents" that allow for change in a networked environment will assist to make this intention a reality [6]. Non-fraudulent mistakes require an approach that provides the corrected information to the widest audience in the most effective and efficient way. Traditional approaches to correcting articles are insufficient to meet these expectations.

We at BioMed Central believe that by providing immediate and unrestricted access, open access online publishing provides a unique environment in which to prevent the perpetuation of errors. To date, we have followed a policy of displaying a note about corrections prominently at the beginning of the original article, in accordance with the National Library of Medicine (NLM) guidelines [7]. In the interests of high quality publication of correct scientific research, we are considering a policy to directly correct our online publications when we have sufficient evidence that the original published article warrants amendment, along with an explanation of how the amended article differs from the original. These updated publications will be reflected on our mirror sites as well.

Of course, it will be up to our authors and our readers to provide a cogent case for the original article to be amended. This argument may also be published as an accompanying commentary to explain the rationale behind the changes that have been made. In cases of fraudulent work, misconduct, or complete invalidation of results, our current policy of retracting an article will remain, in keeping with the NLM guidelines.

We invite our authors, reviewers and readers to join in this debate and to voice your opinions on this proposal by sending us your comments.

## Competing interests

MLN and DCS are employees of BioMed Central and receive fixed salaries.

## Authors' contributions

MLN and DCS contributed equally to the writing of this editorial.

## Pre-publication history

The pre-publication history for this paper can be accessed here:


